# Vegetable oil-based surfactants are adjuvants that enhance the efficacy of neonicotinoid insecticides and can bias susceptibility testing in adult mosquitoes

**DOI:** 10.1371/journal.pntd.0011737

**Published:** 2023-11-17

**Authors:** Fred A. Ashu, Caroline Fouet, Marilene M. Ambadiang, Véronique Penlap-Beng, Colince Kamdem

**Affiliations:** 1 Centre for Research in Infectious Diseases (CRID), Yaoundé, Cameroon; 2 Department of Biochemistry, Faculty of Science, University of Yaoundé, Yaoundé, Cameroon; 3 Department of Biological Sciences, The University of Texas at El Paso, El Paso, Texas, United States of America; Texas A&M University, UNITED STATES

## Abstract

**Background:**

The standard operating procedure for testing the susceptibility of adult mosquitoes to neonicotinoid or butenolide insecticides recommends using a vegetable oil ester (Mero) as a surfactant. However, there is growing evidence that this adjuvant contains surfactants that can enhance insecticide activity, mask resistance and bias the bioassay.

**Methodology/Principal findings:**

Using standard bioassays, we tested the effects of commercial formulations of vegetable oil-based surfactants similar to Mero on the activity of a spectrum of active ingredients including four neonicotinoids (acetamiprid, clothianidin, imidacloprid and thiamethoxam) and two pyrethroids (permethrin and deltamethrin). We found that three different brands of linseed oil soap used as cleaning products drastically enhanced neonicotinoid activity in *Anopheles* mosquitoes. At 1% (v/v), the surfactant reduced the median lethal concentration, LC_50_, of clothianidin more than 10-fold both in susceptible and in resistant populations of *Anopheles gambiae*. At 1% or 0.5% (v/v), linseed oil soap restored the susceptibility of adult mosquitoes fully to clothianidin, thiamethoxam and imidacloprid and partially to acetamiprid. By contrast, adding soap to the active ingredient did not significantly affect the level of resistance to permethrin or deltamethrin suggesting that vegetable oil-based surfactants specifically enhance the potency of some classes of insecticides.

**Conclusions/Significance:**

Our findings indicate that surfactants are not inert ingredients, and their use in susceptibility testing may jeopardize the ability to detect resistance. Further research is needed to evaluate the potential, the limitations and the challenges of using some surfactants as adjuvants to enhance the potency of some chemicals applied in mosquito control.

## Author summary

Monitoring the susceptibility of insect populations to chemical insecticides provides vital information for resistance management. New chemicals are being tested and added to the arsenal used for malaria prevention. As a result, the World Health Organisation (WHO) standard operating procedure for testing insecticide susceptibility in adult mosquitoes has been updated to include new active ingredients. To evaluate their susceptibility to clothianidin, a neonicotinoid or to flupyradifurone, a butenolide, adult mosquitoes are not exposed to the active ingredient alone, but to a mixture containing a surfactant which helps to solubilize the pesticide. In the present study, we report that surfactants such as vegetable oil esters or soap can enhance the potency of some active ingredients. Because of this synergistic action, the actual level of susceptibility of the insect populations tested can be largely overestimated. However, the results also reveal that some affordable products such as soaps can enhance the efficacy of neonicotinoids against mosquitoes. Although it remains unclear how soaps can be used as an adjuvant in insecticide formulations applied against mosquitoes, their potential utility warrants further investigations.

## 1. Introduction

To preserve the efficacy of malaria prevention tools such as long-lasting insecticidal nets and indoor residual spraying, it is crucial to develop new chemicals that provide alternatives to existing insecticides whose potency has been degraded by the spread of resistance among mosquito vector populations [1,2]. Evaluating the susceptibility of wild populations to insecticides is a vital step towards the screening of potential candidates and their deployment in control campaigns. Center for Disease Control and prevention (CDC), World Health Organization (WHO) and academic partners have implemented standard procedures for testing the susceptibility of adult and larval mosquito populations to chemical pesticides [3–7]. These procedures generally consist of determining a discriminating concentration of the insecticide by conducting lethal toxicity assays on susceptible strains and then testing the discriminating concentration on wild mosquito populations to assess their susceptibility profiles.

The neonicotinoid clothianidin is one of three new active ingredients whose formulations have been tested and approved for controlling mosquito populations that have developed resistance to insecticides previously used in large-scale malaria control efforts [2,8–11]. Two manufactured products containing clothianidin are pre-qualified for indoor residual spraying by the WHO [12]. These new formulations are SumiShield (Sumitomo Chemical Company, Japan) and Fludora Fusion (Bayer Crop Science, Monheim, Germany), a combination of clothianidin and deltamethrin [13,14].

Two types of test that can be used to monitor the susceptibility of wild anopheline populations to clothianidin: a WHO paper-test, which assesses mortality rates induced in young adult females by filter papers impregnated with SumiShield and a bottle test which evaluates their tolerance to clothianidin dissolved in a solvent [4,5,15]. Early efforts to implement a standard test were challenged by the moderate solubility of clothianidin observed when large quantities of the active ingredient or its formulated products were being diluted in water, ethanol or acetone [15,16]. To circumvent this issue, it has become common to add a surfactant to the insecticide solutions used for bottle bioassays [2,17–19]. The WHO standard operating procedure for testing the susceptibility of adult mosquitoes to clothianidin recommends mixing the active ingredient with a formulation of rapeseed fatty acid esters (Mero, Bayer, Reading, UK) to increase solubility and enhance uptake of the insecticide by the mosquitoes [4]. Depending on the mosquito species tested, a final concentration of 200 or 800 parts per million (ppm) of Mero is used.

By definition, a surfactant acts as a surface-active agent which reduces the surface tension between two molecules allowing them to be more evenly dissolved in the final solution. Based on their chemical composition and how they work, surfactants applied in insect pest management are split into four categories (nonionic, anionic, cationic or amphoteric) [20,21]. Vegetable oil-based surfactants (e.g., Mero) are obtained from esterification or saponification of seed oil or crop oil. They belong to the class of nonionic surfactants which are widely used in agriculture or gardening activities to solubilize, suspend, or disperse active ingredients allowing more pesticide to reach its target. Surfactants and other adjuvants used in pesticide formulations are often referred to as “inert” ingredients, but some can have biological activity on their own or can enhance the toxicity of insecticides, so that they jointly exert a larger effect than predicted—a phenomenon known as synergy [21,22].

A growing body of evidence suggests that Mero can enhance the activity of some insecticides. Lethal concentrations of clothianidin, acetamiprid, imidacloprid or broflanilide are significantly reduced when a mixture insecticide-Mero is used against *Anopheles* mosquitoes compared to when the active ingredient is tested alone [2,11,19,23–25]. Additionally, studies have shown that a blend containing 800 ppm of Mero and a diagnostic concentration of clothianidin, imidacloprid or acetamiprid induced significantly higher mortality rates in wild populations of *An*. *gambiae* and *An*. *funestus* than the active ingredient alone [18,24]. Importantly, when compared to the standard insecticide synergist pyperonyl butoxide (PBO), Mero enhanced the activity of neonicotinoids more efficiently [24,25]. Taken together, these findings suggest that the synergistic effects of Mero may reduce the sensitivity of insecticide susceptibility tests. Yet, none of the current neonicotinoid formulations developed for indoor residual spraying (SumiShield or Fludora Fusion) contains Mero or other adjuvants designed to enhance their activity against mosquito populations. The example of Mero therefore highlights the unexplored potential role of some surfactants as adjuvant for some public health pesticides. Notably, soaps and other cleaning products that are manufactured in hundreds of cheap formulations contain surfactants with a similar mode of action to Mero. Soaps are interesting candidates to evaluate as potential adjuvants for enhancing the efficacy of some chemicals because they are easy to make using simple ingredients and a basic saponification reaction and are affordable and relatively environmentally friendly.

The aim of the present study was to investigate the effects of linseed oil soap on the potency of clothianidin, acetamiprid, imidacloprid, thiamethoxam, permethrin and deltamethrin. Using concentration-response curves, we established that the surfactant enhanced the toxicity of clothianidin against *An*. *gambiae* mosquitoes. By comparing mortality rates induced by the active ingredient alone or by the insecticide-surfactant mixture in a multiresistant field population of *Anopheles gambiae*, we found that the soap had little effect on resistance to pyrethroids but was an effective adjuvant for neonicotinoids.

## 2. Methods

### 2.1 Ethics statement

Approval to conduct a study in the Center region (N°: 1-140/L/MINSANTE/SG/RDPH-Ce), ethical clearance (N°: 1-141/CRERSH/2020) and research permit (N°: 000133/MINRESI/B00/C00/C10/C13) were granted by the Ministry of Public Health and the Ministry of Scientific Research and Innovation of Cameroon.

### 2.2 Mosquito population

The study focused on *An*. *gambiae* mosquitoes collected from Nkolondom (3°56’43” N, 11°31’01” E) in the suburban area of Yaoundé, Cameroon. We selected this location because larvae and adult *Anopheles* mosquitoes from Nkolondom are highly resistant to neonicotinoids, pyrethroids, organophosphates and carbamates due primarily to chronic exposure to agricultural pesticides [25–31]. Larvae were collected from standing water in furrows between ridges across a large farm in Nkolondom using a dipper [32]. As confirmed by past surveys, anopheline larvae that occur in this farm belong to the nominal species of the *An*. *gambiae* complex: *An*. *gambiae sensu stricto* (hereafter referred to as *An*. *gambiae*, previously known as *An*. *gambiae* S form). After sampling, larvae were transported in plastic containers to the insectary and were reared under standard environmental conditions (25 ± 2°C, 70–80% relative humidity, light: dark cycles of 12:00 h each) while being fed with TetraMin. Adults that emerged were maintained in 30x30x30 cm Bug Dom cages and were provided with cotton pads soaked in 10% sucrose solution. Female adults aged between 2 and 3 days were used for insecticide resistance bioassays. The susceptible strain *An*. *gambiae* Kisumu, which was established in 1975, was reared in the insectary under standard conditions and tested to compare the responses of resistant *vs* susceptible populations.

### 2.3 Insecticides and surfactants

In this study, we used analytical standards of four neonicotinoids (clothianidin, acetamiprid, imidacloprid and thiamethoxam) and two pyrethroids (permethrin and deltamethrin) obtained from Sigma-Aldrich (Pestanal, Sigma-Aldrich, Dorset, United Kingdom). We chose six insecticides for which we had sufficient information on the level of resistance in *Anopheles* mosquitoes collected from Nkolondom. As *An*. *gambiae* larvae and adults from this agricultural site are highly resistant to the six active ingredients tested, this allowed us to assess synergism by detecting any increase in mortality due to the use of a surfactant [25,27,29–31,33]. The insecticides were diluted in absolute ethanol except imidacloprid, which was more soluble in acetone.

The effects of three commercial formulations of liquid linseed oil soap used for surface cleaning were tested. Linseed oil soap is the product of a saponification reaction between linseed fatty acids and potassium hydroxide. The three formulations of liquid soap purchased from supermarkets in Yaoundé, Cameroon, were advertised as being easily biodegradable, made from organic linseed oil and containing 5–30% soap in water and other ingredients such as glycerin and potassium carbonate. The brand names were Maître Savon de Marseille, Saint-Laurent-du-Var, France (hereafter referred to as S1), Carolin Savon noir, Bolton, Belgium (S2) and La perdrix, Paris, France (S3). Insecticide solutions containing soap were prepared by adding the desired volume of S1, S2 or S3 and stored at 4°C for at least 24 h before being used. A solution of ethanol or acetone containing soap was used as control in bioassays.

### 2.4 Bottle bioassays

Insecticide susceptibility was assessed using the WHO bottle bioassay with slight modifications [19]. Wheaton 250-ml bottles and their caps were coated with 1 ml of insecticide solution following the CDC protocol [3]. For each bioassay, we used four test bottles coated with the insecticide and two control bottles coated with the solvent. The discriminating concentration defined as the lowest concentration of the insecticide required to kill 100% of individuals from a susceptible population within 24 h were obtained from published protocols. The following concentrations were tested: 12.5 μg/ml deltamethrin, 21.5 μg/ml permethrin, 75 μg/ml acetamiprid, 150 μg/ml clothianidin, 200 μg/ml imidacloprid and 250 μg/ml thiamethoxam [3,4,23,25]. Control solutions contained either the solvent (ethanol or acetone) or the solvent+soap. To perform bioassay tests, 25 female mosquitoes, 2–3 day old, were aspired from cages and gently released into paper cups and then into the treated bottles for 1 h. The mosquitoes were then transferred into holding paper cups and provided with 10% sugar solution, and mortality was recorded at 24 h. Bioassay testswere conducted under a controlled environment of 25–27°C, 70–90% relative humidity and a 12:12 h light/dark photoperiod.

### 2.5 Surfactant bioassays

To investigate if surfactants can enhance the activity of pesticides, we compared mortality values induced in *An*. *gambiae* mosquitoes exposed either to the insecticide alone or to a mixture containing the active ingredient and linseed oil soap. Mortality would significantly increase in the presence of soap in case of synergistic interactions between the pesticide and the surfactant.

We first tested if three different brands of liquid linseed oil soap could enhance the activity of clothianidin by measuring the mortality rate in the multiresistant *An*. *gambiae* population exposed to a mixture containing 150 μg/ml clothianidin and 1% (v/v) of soap. The standard operating procedure (SOP) for testing adult mosquito susceptibility recommends using 200–800 ppm (0.02–0.08% v/v) of Mero which contains 81% methyl esters of rapeseed oil (surfactant). Since the commercial formulations of linseed oil soap tested were less concentrated (5–30% soap), slightly higher quantities were used (between 0.5 and 1% (v/v)). Prior to the publication of the SOP, 1% Mero was routinely used in bioassays as suggested by the manufacturer [17,18].

We next chose one of the three soap brands that we used to further investigate the interactions between surfactants and pesticides. We tested if halving the concentration of soap or pre-exposing mosquitoes to the surfactant affected the level of susceptibility to clothianidin. We also investigated the effects of 1% (v/v) soap on the level of resistance to four different neonicotinoids and two pyrethroids. Finally, we analyzed the effect of soap on the short-term lethal toxicity of clothianidin using concentration-response curves. If soap enhances the potency of the active ingredient, lethal concentrations would be substantially reduced when a mixture clothianidin-soap is used compared to when the insecticide is applied alone. To test this hypothesis, we subjected female adult mosquitoes to gradual concentrations of clothianidin containing 1% soap and we analyzed the concentration-response curve of the resistant field population and that of the susceptible strain *An*. *gambiae* Kisumu. The following concentrations were tested: *An*. *gambiae* Kisumu (0.39, 0.78, 1.56, 3.12, 4.68, 6.25, 9.37, 12.5, and 25 μg/ml); *An*. *gambiae* Nkolondom (1.56, 3.12, 4.68, 6.25, 12.5, 25, and 100 μg/ml). LC_50_ and LC_99,_ which correspond to the insecticide concentration killing respectively 50% and 99% of exposed individuals were derived within 24 h. The median lethal concentration (LC_50_) for clothianidin alone against *An*. *gambiae* Kisumu was obtained from Agumba *et al* [23] and was compared to the values for the mixture clothianidin-soap.

### 2.6 Data analysis

A log-logistic model was used to fit the concentration-response curve with the *drc* package in R version 4.2.2 based on mortality values obtained from four replicates of 25 mosquitoes each for each insecticide concentration [34]. A probit model was implemented to calculate LC_50_ and LC_99_ with their 95% confidence intervals using the *ecotox* package in R. To quantify the effect of soap on clothianidin toxicity, we computed a synergistic ratio by dividing the LC_50_ value for clothianidin alone to the value for the mixture clothianidin-soap. The higher the ratio, the stronger the effect of soap in enhancing the potency of the active ingredient. The LC_50_ value for clothianidin alone tested against *An*. *gambiae* Kisumu was obtained from [23]. To ascertain if the lethal toxicity of the insecticide-surfactant blend was different between the susceptible strain and the multiresistant filed population, we compared LC_50_ values using a ratio test developed in [35]. We used a Wilcoxon rank sum test to test if there was a significant difference in mortality rates between treatments. All analyses were performed using R version 4.2.2 [34].

## 3. Results

### 3.1 Three different brands of linseed oil soap enhance the potency of clothianidin

**Fig 1:**
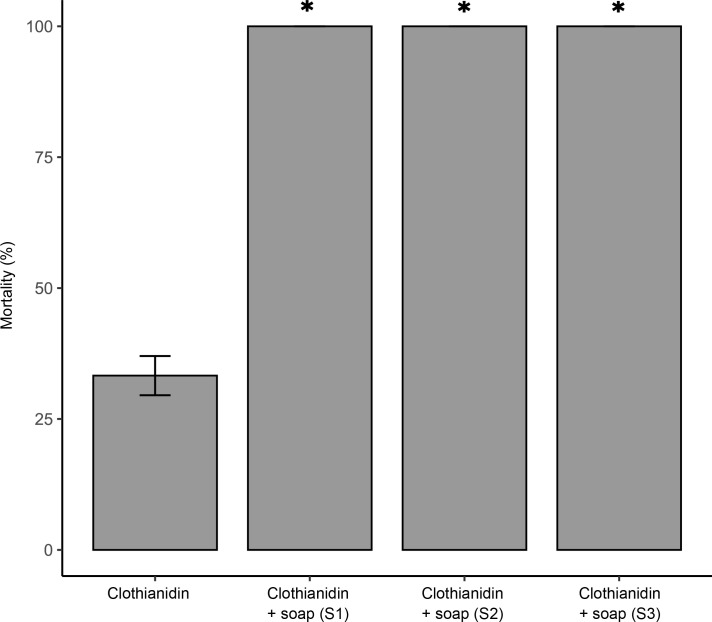
Mortality rates within 24 h of exposure of a resistant *An*. *gambiae* population to clothianidin alone or to a mixture containing 150 μg/ml clothianidin and 1% (v/v) of one of the commercial formulations of linseed oil soap (S1, S2 and S3). Error bars represent the standard error of the mean. * indicates a significant difference in mortality rate between clothianidin alone and a clothianidin-surfactant mixture (Wilcoxon rank sum test, p < 0.05).

To examine if like Mero, other vegetable oil-based surfactants can improve the activity of clothianidin, we tested three different commercial brands of linseed oil soap containing 5–30% surfactant. Two different batches of mosquitoes collected in 2022 and 2023 were tested. The results showed that any of the three brands enhanced the efficacy of clothianidin at a discriminating concentration of 150 μg/ml (Fig 1). The mortality induced in a multiresistant *An*. *gambiae* population sampled from Nkolondom increased from 30 ± 3.49% within 24 h of exposure to clothianidin alone to 100% when 1% (v/v) soap was added to the active ingredient. This test also showed that the synergism between soap and the activity of clothianidin was consistent across soap brands regardless of the difference in surfactant concentration. Moreover, the results were reproducible across two mosquito batches sampled in two different years (2022 and 2023). Mortality varied between 0 and 4% in control tests confirming that, at 1%, soap does not have insecticidal activity against *Anopheles gambiae* adults.

### 3.2 Soap enhanced lethal toxicity of clothianidin

**Fig 2:**
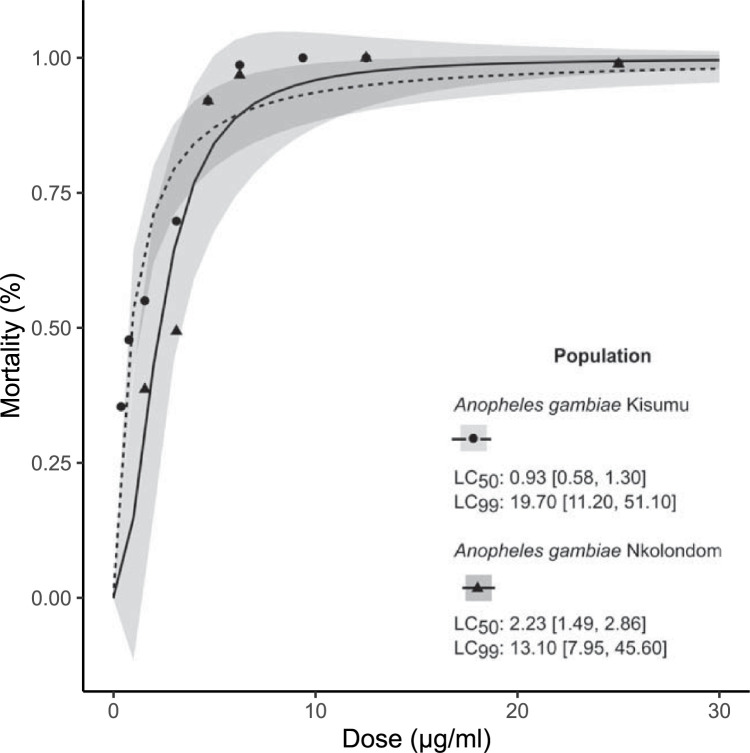
Concentration-response curves with the standard error of the regression model (grey bands) describing the 24-h lethal toxicity of the mixture containing clothianidin (150 μg/ml) and 1% (v/v) of surfactant. LC_50_ and LC_99_ were compared between the susceptible strain *An*. *gambiae* Kisumu and a multiresistant population of *An*. *gambiae* collected from an agricultural area (Nkolondom). A ratio test indicated that there was no significant difference in LC_50s_ between susceptible and resistant mosquitoes when a mixture clothianidin-soap was used (p > 0.05). Each insecticide concentration was tested alongside two controls consisting of 25 adult mosquitoes exposed to bottles coated with 1 ml of absolute ethanol containing 1% soap.

Focusing on clothianidin, we measured to what extent a surfactant can enhance the potency of neonicotinoids. To do so, we tested increasing concentrations of clothianidin containing 1% soap (S1) and we used concentration-response curves and a probit model to evaluate lethal toxicity within 24 h. As indicated by a previous study, when clothianidin is dissolved in ethanol without surfactant, the lethal concentrations, LC_99_ and LC_50_, against the susceptible strain *An*. *gambiae* Kisumu are ~143 μg/mL and 27 μg/ml, respectively [23]. In the present study, we found that adding 1% soap to clothianidin leads to a more than 10-fold reduction in those lethal concentrations (LC_99_: 19.70 μg/ml CI95%[11.20, 51.10] and LC_50_: 0.93 μg/ml [0.58, 1.30]) (Fig 2). In the presence of soap, the short-term lethal toxicity of clothianidin also drastically increased in the multiresistant field population. Within 24 h of exposure, LC_99_ and LC_50_ for the resistant population were 13.10 μg/ml [7.95, 45.60] and 2.23 μg/ml [1.49, 2.86] respectively. The synergistic ratio (i.e., the ratio betweenLC_50_ for clothianidin alone obtained from Agumba et al. and LC_50_ for the clothianidin+surfactant mixture tested in this study) was 29-fold for *An*. *gambiae* Kisumu and 12-fold for *An*. *gambiae* Nkolondom. However, a ratio test indicated that the difference in median lethal concentrations was not statistically significant (p = 0.764) between the resistant and the susceptible population.

### 3.3 The surfactant restored susceptibility to neonicotinoids but not pyrethroids

The analytical standard of clothianidin we tested was soluble in ethanol when the mixture was allowed to rest for at least 24 h, but in general clothianidin is known to be only moderately soluble in ethanol. [23,25,31]. Therefore, the concentration of surfactant which increases the solubility of the active ingredient likely plays a role in the enhanced activity of the clothianidin-soap mixture. To test if reducing the concentration of surfactant affects the mortality rate in resistant mosquitoes, we conducted bioassays adding 0.5% instead of 1% v/v soap (S1) to 8 μg/ml and 15 μg/ml of clothianidin respectively. Both concentrations were chosen within the 95% confidence intervals of LC_99_. Two different batches of 100 mosquitoes collected in 2022 and 2023 were tested. 100% mortality was achieved within 24 h of exposure to each insecticide concentration, which showed that half the quantity of surfactant was sufficient to restore the susceptibility to clothianidin.

In a separate experiment, we first exposed resistant mosquitoes for 1 h in bottles coated with 1% soap dissolved in ethanol (S1) before releasing them for 1 h in bottles containing 8 μg/ml of clothianidin alone. Pre-exposure to soap did not significantly increase mortality (from 30 ± 3.49% to 55 ± 6.60%, Wilcoxon rank sum test, P > 0.05), confirming that soap is more effective as a surfactant mixed with the neonicotinoid insecticide.

**Fig 3:**
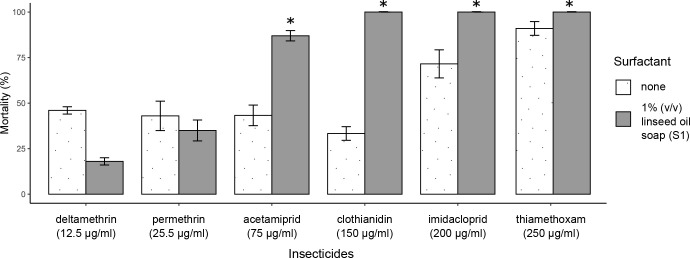
24-h mortality rates (with standard errors of the mean) induced in a multiresistant *An*. *gambiae* population by six different active ingredients alone or in combination with 1% linseed oil soap. The discriminating concentrations used are shown in brackets. Susceptibility of *An*. *gambiae* Kisumu to each discriminating concentration was confirmed. * indicates a significant difference in mortality rates between the test using the active ingredient alone and the bioassay involving a mixture of the insecticide and a surfactant (linseed oil soap) (Wilcoxon rank sum test, p < 0.05).

To determine if vegetable oil-based surfactants induce broad-spectrum synergism beyond improving clothianidin solubility, we tested the effect of linseed oil soap on the activity of other insecticides with different chemical properties and solubility in aqueous solutions. We first measured the baseline susceptibility of the Nkolondom mosquito population to imidacloprid, acetamiprid, thiamethoxam, permethrin and deltamethrin. We then compared mortality rates between tests that used the active ingredient alone and bioassays involving a mixture of the insecticide and 1% (v/v) soap (S1). Fig 3 shows that susceptibility (100% mortality) to thiamethoxam and imidacloprid was restored within 24 h of exposure to the insecticide-soap mixture, while mortality to acetamiprid increased from 43 ± 5.63% to 89 ± 3.25% (Wilcoxon rank sum test, p < 0.05). Meanwhile when linseed oil soap was added to deltamethrin or permethrin, a slight but not significant reduction in mortality was observed (Wilcoxon rank sum test, p > 0.05) suggesting that there was no synergism between this surfactant and the two pyrethroids. Taken together, the results indicate that surfactants contained in some cleaning products may act as adjuvants which enhance the activity of neonicotinoids against *An*. *gambiae* mosquitoes but have little effect on the efficacy of some pyrethroids.

## 4. Discussion

Searching for new chemicals is vital for mitigating the effects of mosquito adaptation to insecticides and for upholding the progresses made in malaria prevention [11,12]. Some formulations of agrochemicals with new modes of action have revealed promising results in field trials and could help reduce the negative impacts of insecticide resistance [14,36–38].

In the current study, we have shown that linseed oil soap increases the susceptibility of *An*. *gambiae* mosquitoes to neonicotinoids including clothianidin whose formulations have been prequalified for indoor residual spraying [12–14]. Our control experiments where female adults were exposed to linseed oil soap alone allowed us to rule out the possibility that this surfactant on its own had lethal effects on mosquitoes. Corbel et al. [19] have also revealed that, at 200 or 800 ppm, Mero, a formulation of rapeseed fatty acid esters, had no insecticidal activity against *Anopheles* mosquitoes. Some soaps have often been used as an insecticide in gardening activities to control some plant pests, the most susceptible being small, soft-bodied arthropods such as aphids, mealybugs, psyllids and spider mites [39,40]. Our results indicate that adult mosquitoes are not susceptible to soap at concentrations lesser than 1%.

Using estimates of 24-h LC_50_ and LC_99_, we found that blends of clothianidin and linseed oil soap are highly potent leading to a drastic increase in short-term lethal toxicity of the active ingredient. The lethal concentrations detected in our study are in the range of values obtained when concentration-response curves of *An*. *gambiae* mosquitoes are established using clothianidin mixed with Mero (0.02–1%) [2,19]. According to LC_50_, the combined effects of clothianidin and soap are more than 10-fold higher than that of clothianidin alone. The effect was more pronounced in susceptible compared to resistant populations (synergistic ratio: 29-fold vs 12-fold) although a ratio test did not reveal a significant difference in LC_50s_. These synergistic ratios are comparable to values detected with some compounds having strong synergistic interactions with the insecticide [22,41].

The mixture between 1% linseed oil soap and clothianidin restored neonicotinoid susceptibility in a multiresistant *An*. *gambiae* population collected from an agricultural area. Similarly, a recent study has found that 100% mortality was achieved when adults from the same population were exposed to a blend containing 150 μg/ml of clothianidin and 1% Mero [18]. We did not conduct a detailed analysis of the effect of the surfactant concentration on insecticide activity, but we observed that the efficacy of clothianidin was restored with 1% or 0.5% soap. These results corroborate previous tests indicating that Mero can enhance the potency of clothianidin in *Anopheles* mosquitoes even at relatively low concentrations [19]. Indeed, at 0.08% v/v, Mero drastically increases the activity of neonicotinoid insecticides including clothianidin, acetamiprid and imidacloprid against *An*. *funestus* [24]. Similarly, we observed that 1% linseed oil soap enhanced the potency of other neonicotinoid insecticides as surfactant-insecticide mixtures restored the susceptibility of the multiresistant *An*. *gambiae* population to thiamethoxam, imidacloprid and to a lesser extend acetamiprid. In previous studies, it has been shown that the cytochrome inhibitor piperonyl butoxide (PBO) only partially restored susceptibility to acetamiprid, clothianidin and imidacloprid in *An*. *funestus* [24]. A comparable result was obtained in *An*. *gambiae* at the exception of acetamiprid whose efficacy was fully restored in some populations [25,31]. Comparatively, vegetable oil-based surfactants are therefore more effective than the standard synergist PBO in enhancing the efficacy of neonicotinoids in laboratory bioassays.

Significant research has provided examples of synergistic interactions between adjuvants and neonicotinoids. Notably, some agricultural spray adjuvants have been shown to increase the toxicity of acetamiprid against honeybees [42]. Alkyl phenoxy polyethylene ethanol used as adjuvant in thiamethoxam formulations against the whitefly *Bemisia tabaci* resulted in 25-fold increase in the potency of the mixture compared to that of the active ingredient alone [43]. Another investigation indicated that a vegetable oil-based adjuvant had synergistic effects resulting in increased efficacy of thiamethoxam against cowpea thrips [44]. More generally, a review of research comparing insecticide formulations versus the active ingredient concluded that the presence of adjuvants in the formulations, in most cases, results in increased toxicity of the product formulations versus their active ingredients [20]. Overall, there is sufficient evidence, including findings from our study, that adjuvants such as surfactants can inflate the activity of chemicals leading to an overestimation of their actual efficacy against insects. This overestimation may cause bias in estimating and interpreting insecticide susceptibility in wild populations. Although the mechanisms of synergy between surfactants and neonicotinoids remain unclear, it is likely that increased solubility and uptake of the active ingredient by adult mosquitoes is a major contributing factor. Notably, some organosilicon surfactants used in agricultural pest management are believed to modify the surface tension of plant cells and insect cuticles thereby increasing the penetration of neonicotinoid insecticides [45].

The standard operating procedure for testing the susceptibility of adult mosquitoes recommends using 200–800 ppm Mero as a surfactant when evaluating the activity of neonicotinoids and butenolides against *Anopheles* and *Aedes* populations [4,19]. Yet mounting evidence suggests that Mero can synergize the activity of some insecticides including neonicotinoids and broflanilide, a new meta-diamide insecticide [11,18,24]. For such new insecticides, it is important to rely on sensitive tests that can detect early stages of resistance when effective management efforts can successfully mitigate its negative impacts. This objective would be difficult to achieve with standard tests that use extremely potent mixtures of the active ingredient and a synergist. It is therefore vital to identify the insecticides or classes of insecticides for which vegetable oil-based surfactants may not be used in susceptibility testing due to potential synergic interactions. In this study, we have tested if soap could enhance the activity of pyrethroids that are very different from neonicotinoids based on chemical characteristics and mode of action. We observed that soap did not significantly affect the efficacy of deltamethrin and permethrin. As opposed to the synergistic effects of some surfactants on neonicotinoids, which have been relatively well studied, the interactions between vegetable oil-based surfactants and pyrethroids remain largely unexplored. The difference observed in this study between neonicotinoids and pyrethroids is probably due to the chemical properties of each class of insecticides. Neonicotinoids are highly water soluble compared to pyrethroids that are only moderately soluble in aqueous solutions [9]. However, in comparison to pyrethroids, neonicotinoids probably have lower binding affinity to organic materials, and as a result, when their surface tension is reduced with a surfactant, their uptake and potency increase [20].

Standard tests conducted on larval and adult populations showed that some neonicotinoids may have suboptimal activity against *Anopheles* mosquitoes [1,17,23,25,30,31]. Most active ingredients in this class of insecticides have substantially higher lethal concentration compared with some public health insecticides [1,16,17,23,25]. In addition, bioassays have revealed reduced susceptibility to acetamiprid, thiamethoxam, imidacloprid and clothianidin in some wild populations of *An*. *gambiae* and *An*. *coluzzii* raising concern about the potential efficacy of neonicotinoids against malaria vectors [15,17,25,30,31]. Our results show that beside PBO, the utility of products such as soap that are affordable, available and easy to make as adjuvant for neonicotinoids could be further investigated. Recently, there has been increasing interest in the use of synergists in malaria vector control, and at least one commercial formulation of long-lasting insecticidal net contains PBO combined to a pyrethroid [46]. Although it remains unclear how surfactants can be used with vector control tools such as long-lasting insecticidal nets and indoor residual spraying, the present study shows that the potential of soaps as an insecticide adjuvant is worthy of future research.

In conclusion, our study reveals that surfactants can have both negative and positive implications for insecticide resistance management depending on how they are used. The effects of surfactants as an activity enhancer can undermine early detection and management of resistance to some classes of insecticides whereas the same effects can on the contrary be beneficial to the efficacy of some chemicals. Future research directions include testing a concentration gradient of pure soap and conducting a broader assessment of adjuvants used in susceptibility tests such as silicon oil which is applied while testing pyrethroids with WHO paper tests.
